# Myostatin Deficiency Enhances Antioxidant Capacity of Bovine Muscle via the SMAD-AMPK-G6PD Pathway

**DOI:** 10.1155/2022/3497644

**Published:** 2022-05-25

**Authors:** Lin Zhu, Xueqiao Wang, Zhuying Wei, Miaomiao Yang, Xinyu Zhou, Jiaru Lei, Chunling Bai, Guanghua Su, Xuefei Liu, Lei Yang, Guangpeng Li

**Affiliations:** State Key Laboratory of Reproductive Regulation and Breeding of Grassland Livestock, Inner Mongolia University, Hohhot 010070, China

## Abstract

During exercise, the body's organs and skeletal muscles produce reactive oxygen species (ROS). Excessive ROS can destroy cellular lipids, sugars, proteins, and nucleotides and lead to cancer. The production of nicotinamide adenine dinucleotide phosphate (NADPH) by the pentose phosphate pathway (PPP) is an auxiliary process of the cellular antioxidant system that supplements the reducing power of glutathione (GSH) to eliminate ROS in the cell. Myostatin (MSTN) is mainly expressed in skeletal muscle and participates in the regulation of skeletal muscle growth and development. Loss of MSTN leads to muscular hypertrophy, and MSTN deficiency upregulates glycolysis. However, the effect of MSTN on the PPP has not been reported. This study investigated the effect of MSTN on muscle antioxidant capacity from a metabolic perspective. We found that reducing MSTN modulates AMP-activated protein kinase (AMPK), a key molecule in cellular energy metabolism that directly regulates glucose metabolism through phosphorylation. Downregulation of MSTN promotes tyrosine modification of glucose-6-phosphate-dehydrogenase (G6PD) by AMPK and is regulated by the Smad signaling pathway. The Smad2/3 complex acts as a transcription factor to inhibit the AMPK expression. These results suggest that reduced MSTN expression inhibits the Smad signaling pathway, promotes AMPK expression, enhances the activity of G6PD enzyme, and enhances the antioxidant capacity of nonenzymatic GSH.

## 1. Introduction

MSTN is a member of the transforming growth factor-*β* superfamily that functions as a negative regulator of skeletal muscle mass. Inhibition of MSTN and knockdown of the MSTN gene cause muscle hypertrophy, which results in ‘double-muscled' cattle, sheep, mice, rabbits, dogs, and humans [1]. MSTN is predominately expressed in skeletal muscles but is also expressed in other tissues [2, 3]. As a signaling ligand, MSTN modulates muscle mass development through the MSTN-activin receptor IIB- (ActRIIB-) Smad2/3 signaling pathway [4, 5]. Basic metabolic changes under the influence of *MSTN* indicate that MSTN plays an important role in the regulation of energy metabolism. In C2C12 cells, overexpression of MSTN results in decreased glucose transporter type 4 (*Glut4*) gene expression and glucose uptake, as well as decreased active phosphorylation of insulin receptor substrate 1 (IRS-1) and phosphoinositide 3-kinase (PI3K), decreased phosphorylation of AKT, and increased phosphorylation of glycogen synthase kinase 3*β* (GSK3*β*) [6]. Treatment with antimyostatin antibody increases insulin-stimulated glucose metabolism in elderly mice, possibly due to increased glucose uptake in insulin-stimulated skeletal muscle [7]. MSTN inhibits glycolysis and increases glycogen accumulation [8]. In *MSTN^−/−^* mice, enolase activity, a key component of glycolysis, increases along with serum lactate level in the resting state and after exercise, and the soleus muscle changes from the “slow oxidative” type to the “fast glycolytic” type [9]. In contrast, in the presence of 0.1–1.5 *μ*g/ml recombinant MSTN, glycolysis in C2C12 cells is increased, and glycogen synthesis is inhibited [10]. MSTN seems to regulate glycolysis and glycogen synthesis through the AMPK pathway [8].

The pentose phosphate pathway (PPP) is a way of oxidative decomposition of glucose. The PPP reaction system starts with glucose-6-phosphate, which produces pentose, inorganic phosphoric acid, and NADPH in oxidative decomposition. NADPH can provide reducing capacity for GSH and enhance antioxidant capacity [11, 12]. G6PD, the first rate-limiting PPP enzyme, controls NADPH production [13] and modulates the cytoplasmic redox state [14].

AMPK is a cellular energy status sensor and key regulator of mitochondrial function and oxidative stress in skeletal muscle. AMPK*γ*3 can affect phosphorylation of AMPK1/2 and prevent dephosphorylation of protein phosphatase Thr172 [15]. MSTN affects protein and fat metabolism by inhibiting the AMPK signaling pathway. Low concentrations of MSTN can inhibit the eukaryotic elongation factor-2 kinase (eEF2K)/eukaryotic elongation factor-2 (eEF2) pathway through AMPK signals and suppress protein synthesis, but low MSTN concentration does not affect the mTOR pathway [16]. Blocking MSTN with soluble activin receptor IIb (sActRIIB-Fc) enhances mTOR complex 1 (mTORC1) signaling, increases regulated in development and DNA damage responses 1 (REDD1) protein and eukaryotic translation initiation factor 2B subunit epsilon (eIF2B*ε*) protein, and improves phosphorylation of eukaryotic translation initiation factor 4E-binding protein 1 (4E-BP1) and AMPK [17]. Loss of MSTN suppresses fat accumulation with a 70% reduction in total body fat and approximately 25% and 40% reductions in gonadal and genital fat pads, respectively [18]. Molecular evidence suggests that MSTN mutations increase fatty acid oxidation and enhance brown fat formation by increasing lipolysis activity [19]. The transformation of white adipose tissue to brown adipose tissue is mediated by peroxisome proliferator-activated receptor *γ* coactivator-1*α* (PGC1*α*-irisin) [20] and the AMPK-PGC1*α*-fibronectin type III domain-containing protein 5 (Fndc5) pathway [21].

Although the contribution of MSTN to glycolysis has been extensively analyzed, similar analyses of the PPP and NADPH metabolism are lacking. In this study, we demonstrate that downregulation of MSTN enhances the antioxidant capacity of bovine muscle through PPP and Smad-AMPK-G6PD axis.

## 2. Materials and Methods

### 2.1. Cattle

We used CRISPR/Cas9 and somatic cell nuclear transfer to generate MSTN knockout cattle. The *MSTN^+/-^* cattle were the offspring of *MSTN^−/−^* bulls bred with wild-type (WT) cows [22, 23]. Selected *MSTN^+/-^* (MT) and WT 24-month-old heifers were used in this study. All heifers were fed in the same pasture with the same feeding consistency. All experiments were performed in strict accordance with the guidelines of the Experimental Animal Management and Operation Standards of Inner Mongolia University (no. IMU-CATTLE-2020-034, 01 June 2020).

### 2.2. Cattle Muscle Satellite Cell Culture

The longissimus dorsi muscles were dissected and cut into pieces. The cut tissue pieces were immersed in 75% alcohol for 10 s, followed by 3 immersions in PBS (FG701-01, TRANS, China) containing 2% Pen Strep (15140-122, Gibco, USA) for 20 s. The muscle pieces were then soaked in DMEM F12 (11330107, Gibco, USA), 20% FBS (10099141, Gibco, USA), 10% HS (S9050, Solarbio, China), and Pen Strep- for 10 s. Then, the tissue pieces were placed upside down in a 100 mm dish in an incubator (5% CO_2_, 38.5°C) for 2 h. Seven to eight milliliters of culture medium were added to the dishes, and the tissue was cultured until satellite cells appeared.

### 2.3. Quantitative Real-Time PCR

Total RNA was isolated from tissues using the RNAiso Plus kit (9109, Takara, Japan) according to the manufacturer's instructions. The RNA was reverse transcribed into cDNA using a cDNA reverse transcription kit (RR036A, Takara, Japan). All primer sequences are summarized in Table S1. Real-time PCR (qPCR) was performed using a real-time PCR detection system (7500, ABI, USA). The reaction mixture (20 *μ*l) contained 1 *μ*l cDNA, 0.5 *μ*l of each primer, and 10 *μ*l TB Green Supermix (RR820A, Takara, Japan). After the initial denaturation step at 95°C for 30 s, 40 cycles at 95°C for 15 s and 60°C for 30 s were performed. Gene expression was detected by 2^−ΔΔCT^ relative quantitative analysis. All experiments were repeated more than three times.

### 2.4. Western Blotting

Proteins were extracted from tissues and cells with cell lysis buffer, boiled for 5 min, and stored at -80°C until use. Samples were separated using 10% sodium dodecyl sulfate-polyacrylamide gel electrophoresis (SDS-PAGE) and electrically transferred to polyvinylidene fluoride (PVDF) membranes blocked with 5% nonfat dry milk in TBS-Tween (blocking buffer) for 1 h. PVDF membranes were probed at 4°C overnight in blocking buffer with the following primary antibodies: MSTN (ab201954, Abcam, USA), p-Smad2 (ab280888, Abcam, USA), p-Smad3 (ab52903, Abcam, USA), Smad2 + Smad3 (ab202445, Abcam, USA), GLUT1 (A11727, ABclone, China), GLUT4 (A7637, ABclone, China), p-AMPK*α*1+*α*2 (ab133448, Abcam, USA), G6PD (ab993, Abcam, USA), TKL (1 : 1000, ab181235, Abcam, USA), RPI (ab137629, Abcam, USA), phosphoserine (ab9332, Abcam, USA), phosphotyrosine (ab10321, Abcam, US), p-AKT (ab38449, Abcam, USA), p-P38 (ab31828, Abcam, USA), and *α*-tubulin (11224-AP, Proteinch, China). Membranes were washed three times and incubated with secondary antibody diluted 1 : 5000 in blocking buffer. Finally, protein expression was detected and recorded.

### 2.5. Metabolic Substrate and Enzyme Assays

Commercial kits (COMIN Biotechnology, China) were used to detect hexokinase (HK) (HK-1-Y), phosphofructokinase (PFK) (PFK-1-Y), triosephosphate isomerase (TPI) (TPI-1-G), pyruvate kinase (PK) (PK-1-Y), FDP (FDP-1-G), pyruvate (PA) (PA-1-Y), lactic acid (LA) (LA-1-G), G6PD (G6PDH-1-Y), NADP^+^/NADPH (NADP-1-Y), ROS (ROS-1-Y), H_2_O_2_ (H_2_O_2_-1-Y), malondialdehyde (MDA) (MDA-1-Y), superoxide dismutase (SOD) (SOD-1-Y), catalase (CAT) (CAT-1-Y), glutathione peroxidase (GSH-PX) (GPX-1-W), GR (GR-1-W), glutathion (GSH) (GSH-1-W), reduced glutathione (GSSG) (GSSG-1-W), mitochondrial respiratory chain complex I and III, (FHTA-2-Y, FHTC-2-Y), Na^+^-K^+^ pump (NKATP-1-Y), Ca^2+^-Mg^2+^ pump (CMATP-1-Y), and glycogen (TY-1-Y). Samples of 0.1 g muscles were analyzed following the manufacturer's instructions. Briefly, 0.1 g tissue was placed into a homogenizing tube containing 1 ml extract from the kit along with about 15 ceramic beads. The tissue was homogenized at low temperature with a homogenizer (Bertin, France). The supernatant was collected and added into 96-well plate with other reagents according to the instructions. After reading the absorbance value on the microplate spectrophotometer (Thermo, USA), the enzyme activity or metabolite concentration was calculated by formula according to the instructions.

### 2.6. Transcriptome Analysis of Muscles

Total RNA was extracted with TRIzol reagent (Invitrogen, CA, USA). Poly(A) RNA was purified from total RNA (5 *μ*g) by poly-T oligo-attached magnetic beads and fragmented into small pieces. The spliced RNA fragments were reverse transcribed to construct the final cDNA library, and paired-end sequencing was performed on IlluminaHiseq4000 (LC Sciences, USA). The read values were aligned with the UCSC (http://genome.ucsc.edu/) reference genome using the HISAT package. A portion of the reads were deleted based on the quality of information accompanying each read, and the remaining reads were mapped to the reference genome. All transcriptomes from samples were merged to reconstruct a comprehensive transcriptome using the Perl scripts StringTie and edgeR. Differentially expressed mRNAs and genes with log2 (fold change) > 1 or log2 (fold change) < −1 and *p* value < 0.05 were screened by the R package. Transcriptome data can be found at this link (https://www.ncbi.nlm.nih.gov/geo/info/linking.html).

### 2.7. Chromatin Immunoprecipitation-qPCR Assay

Target gene promoter region sequences (2000 bp upstream of the transcription initiation site) were used to predict Smad2 : Smad3 : Smad4 transcription factor binding sites (http://jaspar.genereg.net/analysis). PCR primers were designed according to the predicted binding site of transcription factors, as shown in Table S2.

Chromatin immunoprecipitation (ChIP) assays were performed according to the manufacturer's instructions (26157, Thermo, USA). Briefly, approximately 0.2 g tissues were fixed with 1% formaldehyde and quenched by glycine. The tissues were washed 3 times with PBS and harvested in ice-cold PBS containing 1% Halt Cocktail. DNA was lysed and digested by MNase and then sonicated on ice with pulses to break the nuclear membrane. The lysates were incubated with anti-Smad2+Smad3 antibody (ab202445, Abcam, USA) and protein G beads overnight at 4°C. Normal rabbit IgG was used as a negative control. The DNA was eluted 4 times with a ChIP elution buffer, and the eluent was incubated at 65°C for 1.5 h. DNA was recovered using a DNA purification kit, and the purified DNA was assayed by qPCR.

### 2.8. Coimmunoprecipitation

The coimmunoprecipitation (Co-IP) process followed the instructions of the Pierce classic IP kit (26146, Thermo, USA). To lyse the muscle tissues and cells, 1 ml ice-cold IP lysis buffer was added to the tissues and cells, which were incubated on ice for 5 min with periodic mixing. The supernatant was transferred to a new tube, and 1 ml of lysate was prepurified with 80 *μ*l agarose resin. Ten micrograms of affinity-purified antibody were combined with 600 *μ*l precleared cell lysate overnight in a 4°C microcentrifuge tube to form an immune complex. The immune complex was captured with protein A/G and agarose in the spin column and washed three times with 200 *μ*l washing buffer. The immune complexes were eluted with sample buffer and incubated at room temperature for 5–10 min, and the eluents were collected by centrifugation (10000 g). Samples were left at -20°C prior to SDS-PAGE gel application.

### 2.9. Statistical Analyses

All data were expressed as the mean ± SD of at least three independent trials. In the graphs, bars represent means, while error bars represent one standard deviation. Welch's two-tailed *t*-test was used for statistical analysis when data from two groups with different standard deviations were compared. When comparing multiple groups, a repeatable one-way ANOVA was performed, followed by a post hoc analysis using Bonferroni's correction to adjust for multiple comparisons. ^∗^*p* < 0.05 and ^∗∗^*p* < 0.01 were considered statistically significant. The histograms were drawn in Prism 7.0 (GraphPad, USA), and phototypesetting was done with Adobe Photoshop CS3.

## 3. Results

### 3.1. The Reduction of MSTN Alters Muscle Transcriptome

**Figure 1:**
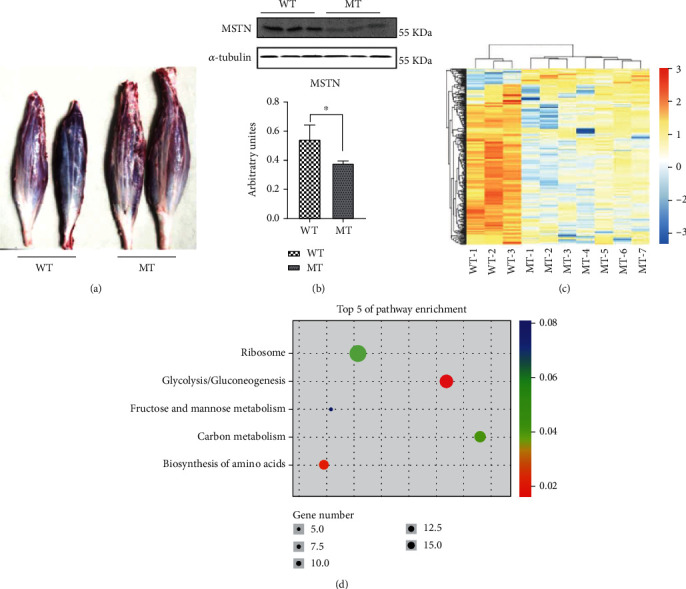
The reduction of of MSTN alters muscle transcriptome. (a) The morphology of soleus muscles. (b) Western blot detection of MSTN protein in cattle muscle. (c) Cluster analysis of differential gene expression. (d) KEGG enrichment analysis of upregulated genes. MT: MSTN knockout cattle group; WT: wild-type cattle group. Data presented are means ± SD. One-way ANOVA with post hoc LSD multiple comparison test. ^∗^*p* < 0.05, ^∗∗^*p* < 0.01.

The soleus muscle had lesser mass in WT cattle compared to MT cattle (Figure 1(a)). Accordingly, the concentration of MSTN protein per volume of plasma in MT cattle was only 22.11 ± 1.09 ng/ml, which was significantly lower than in WT cattle 38.92 ± 3.48 ng/ml (*p* < 0.01, Figure S1A). The MSTN were also downregulated in MT cattle muscles (Figure 1(b)). Transcriptome sequencing analysis of seven MT cattle and three WT cattle showed significant differences in gene expression between the MT and WT cattle (Figure 1(c)). A total of 342 differentially expressed genes were identified, of which 312 were downregulated and 30 upregulated in MT muscle (Figure 1(c)). KEGG enrichment analysis was performed on metabolic pathways. Upregulated genes included genes involved in glycolysis and gluconeogenesis, biosynthesis of amino acids, carbon metabolism, and fructose and mannose metabolism (Figure 1(d)).

### 3.2. The Reduction of MSTN Promotes Glucose Catabolism

**Figure 2:**
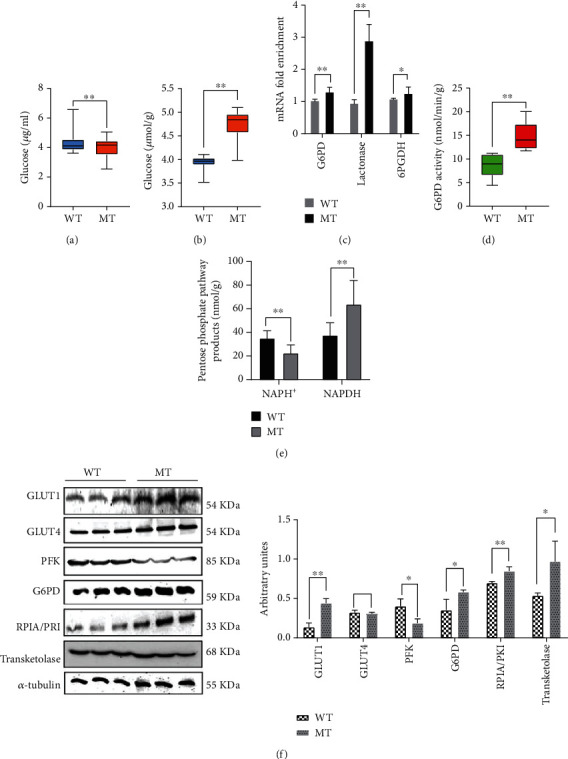
The reduction of MSTN promotes glucose catabolism. (a) The concentration of glucose in plasma. (b) The concentration of glucose in the muscle. (c) mRNA expression of pentose phosphate pathway genes in the oxidation-limited stage of muscle. (d) The activity of G6PD in cattle muscles. (e) The concentration of NADP^+^ and NADPH in cattle muscle. (f) The protein expression of PFK, GLUT1, and GLUT4 in muscle; the protein expression of G6PD, RPI, and TKL in muscle. MT: MSTN knockout cattle group; WT: wild-type cattle group. Data presented are means ± SD. One-way ANOVA with post hoc LSD multiple comparison test. ^∗^*p* < 0.05, ^∗∗^*p* < 0.01.

The transcriptome data indicate that most of the upregulated genes are associated with glucose metabolism. To determine the effect of mutating MSTN on glucose metabolism, the levels and activity of products and enzymes involved in glucose catabolism were measured. MT cattle had significantly lower blood glucose than WT cattle (Figure 2(a)). However, muscle glucose levels were significantly higher in the MT group than in the WT group (Figure 2(b)). The expression of the glucose transporter gene *GLUT1* is increased in MT cattle, while *GLUT4* expression is similar to WT cattle (Figure 2(f)). The content of insulin, the regulator of glucose transporters, did not differ between MT and WT cattle (Figure S2A). These results imply that more blood glucose enters muscle cells in the MT cattle vs. WT cattle.

During glycolysis, the activities of most rate-limiting enzymes are increased. Accordingly, hexokinase (HK) and pyruvate kinase (PK) were significantly higher in MT cattle than in WT cattle (Table 1). Phosphofructokinase (PFK), the most important rate-limiting enzyme in glycolysis, was significantly lower in MT cattle (144.10 ± 15.01 nmol/min/g) than in WT cattle (193.8 ± 14.30 nmol/min/g) (Table 1). PFK protein in the MT group was also lower than the WT group (Figure 2(f)). Fructose-1,6-diphosphate (FDP), a product of PFK acting on fructose 6-phosphate, was significantly lower in MT cattle (3.90 ± 0.33 mg/g) compared to WT cattle (6.07 ± 0.53 mg/g) (*p* < 0.01, Table 2). Triosephosphate isomerase (TPI), the conversion enzyme between dihydroxyacetone phosphate and glyceraldehyde-3-phosphate, was significantly lower in MT cattle muscle (Table 1), and the concentration of pyruvate (PA) in muscle and plasma was significantly higher (*p* < 0.01, Table 2, Figure S2B). The lactic acid (LA) content in MT cattle muscle was significantly upregulated (Table 2), but we saw no significant difference in the content of LA in plasma between MT and WT cattle (Figure S2C).

Muscular glucose catabolism involves glycolysis and the pentose phosphate pathway. The mRNA of key enzymes such as phosphoglucose dehydrogenase (G6PD), lactonase, and 6-phosphogluconate dehydrogenase (6PGDH) in the oxidation stage of the PPP were more highly expressed in MT cattle (Figure 2(c)). The enzymatic activity of G6PD was significantly higher in MT cattle compared to WT cattle (14.74 ± 0.79 vs. 8.79 ± 1.20 nmol/min/g), and the protein content was similarly increased (Figures 2(d) and 2(f)). Muscle NADPH concentration, the product of the PPP oxidation stage, was significantly higher in MT cattle (63.85 ± 6.3 vs. 37.52 ± 3.15 nmol/g), while NADP^+^ was significantly lower (22.54 ± 2.03 vs. 35.03 ± 1.95 nmol/g) (*p* < 0.01, Figure 2(e)), suggesting that NADP^+^ is more actively converted to NADPH in the PPP in MT vs. WT cattle. At the same time, the concentrations of key enzymes such as ribulose-5-phosphate epimerase (RPI), transketolase (TKL), and MT muscle protein in the nonoxidative phase of the PPP are significantly increased (Figure 2(f)). From the perspective of glycogen synthesis, the glycogen accumulation in the muscle of MT cattle is lower than in WT cattle (Figure S2D). The mRNA expression of glycogen synthase kinase-3*β* (GSK3*β*), which negatively regulates the expression of the GS gene, was significantly increased compared to the control (*p* < 0.01, Figure S2E). Reduced MSTN expression inhibited the first stage of glycolysis, while upregulated PPP provided a substrate for the second stage of glycolysis, which together produced more pyruvate and LA.

### 3.3. The Reduction of MSTN Promotes Muscle Antioxidant Capacity through G6PD in the Pentose Phosphate Pathway

**Figure 3:**
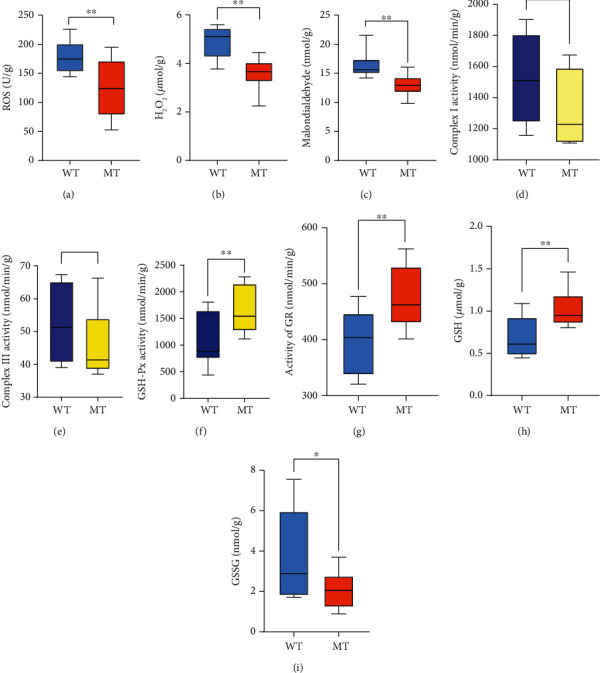
MSTN promotes muscle antioxidant capacity through G6PD in the pentose phosphate pathway. (a) The concentration of oxygen free radical in the muscles. (b) The concentration of H_2_O_2_ in the muscles. (c) The concentration of malondialdehyde in the muscles. (d) The activity of mitochondrial respiratory complex I in the muscles. (e) Activity of mitochondrial respiratory complex III in the muscles. (f) The activity of GSH-Px in the muscles. (g) Activity of GR in the muscles. (h) The concentration of GSH in the muscles. (i) The concentration of GSSG in the muscles. MT: MSTN knockout cattle group; WT: wild-type cattle group. Data presented are means ± SD. One-way ANOVA with post hoc LSD multiple comparison test. ^∗^*p* < 0.05, ^∗∗^*p* < 0.01.

The PPP provides reducing power for antioxidants. Based on the above results, the antioxidant capacity of muscles was studied. The content of oxygen free radicals (ROS) in the muscles of MT cattle was significantly lower than in WT cattle (124.2 ± 11.44 vs. 180.1 ± 9.18 U/g) (*p* < 0.01, Figure 3(a)). The content of H_2_O_2_ (3.61 ± 0.19 vs. 4.91 ± 0.26 *μ*mol/g) and malondialdehyde (MDA) (12.91 ± 0.39 vs. 16.37 ± 0.76 nmol/g) in the muscle were also reduced in MT cattle (Figures 3(b) and 3(c)). Cellular ROS is mainly produced by mitochondrial complex I and complex III. In MT cattle, the activity levels of complex I and complex III did not differ compared to WT cattle (Figures 3(d) and 3(e)). We tested the antioxidants of cattle muscle and found that the antioxidants related to enzymes and GSH were significantly increased in MT cattle. Glutathione peroxidase (GSH-PX), catalyzer of GSH into GSSG, reduces the toxic peroxides to nontoxic hydroxyl compounds. Mutation of MSTN enhanced GSH-PX activity (Figure 3(f)). Glutathione reductase (GR), the enzyme that catalyzes the transformation of glutathione disulfide (GSSG) to GSH, was higher in MT cattle than in WT cattle (Figure 3(g)). Superoxide dismutase (SOD) and catalase (CAT) are the most active members of the enzyme antioxidant system, and there was no significant difference in either between MT and WT cattle (Figures S3A, B). In the nonenzymatic antioxidant system, GSH is an important reducing agent in cells, and NADPH produced by the PPP promotes the production of GSH [11]. In MT cattle, the level of GSH was higher than in WT cattle (1.029 ± 0.06 vs. 0.695 ± 0.08 *μ*mol/g) (*p* < 0.01, Figure 3(h)), as was the GSH/GSSG ratio (Figure S3D). In contrast, GSSG, the oxidated state of GSH, was significantly lower in MT cattle (2.056 ± 0.24 vs. 3.79 ± 0.74 nmol/g) (*p* < 0.05, Figure 3(i)). The activity of superoxide radical scavenging (SRSA) was enhanced in MT cattle (0.22 ± 0.01% vs. 0.14 ± 0.02%) (*p* < 0.01, Figure S3C). ROS damage sodium-potassium (Na^+^-K^+^) pumps and calcium-magnesium (Ca^2+^-Mg^2+^) pumps [24, 25]. The activities of Na^+^-K^+^ pumps and Ca^2+^-Mg^2+^ pumps in MT muscle were both higher than those in the WT cattle muscle (Figures S3E, F). These results suggest that reducing MSTN reduces muscle oxidative damage by increasing GSH.

### 3.4. The Reduction of MSTN Enhanced AMPK Interaction with G6PD

**Figure 4:**
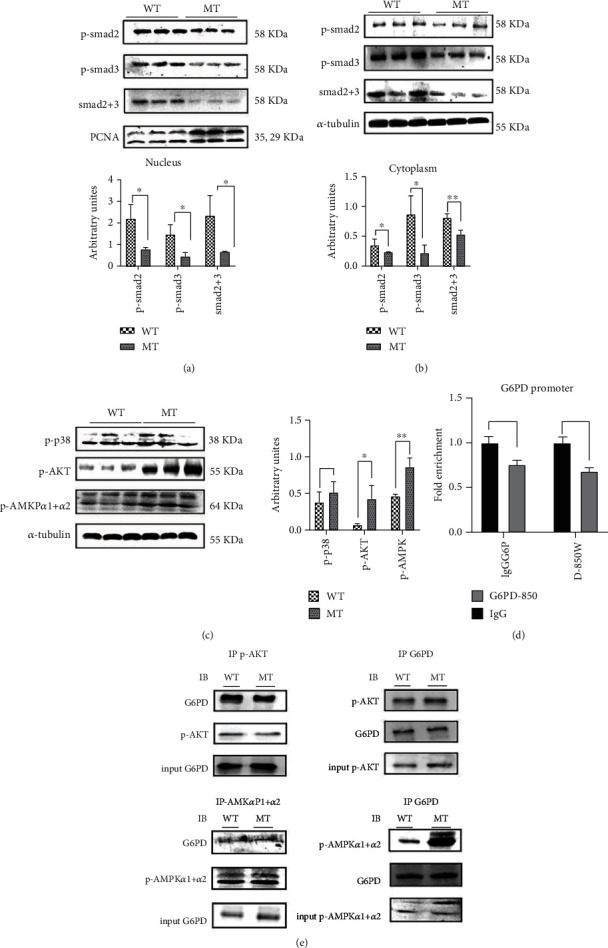
The reduction of MSTN enhanced AMPK interaction with G6PD. (a) p-Smad2/3, Smad2+3 protein content in the nucleus of the cattle muscles. (b) p-Smad2/3, Smad2+3 protein content in cytoplasm of the cattle muscles. (c) The Western blot results of TGF-*β* nonclassical signaling pathway (p-p38, p-AKT), p-AMPK*α*1+*α*2. (d) The binding of Smad2/3 to the GP6D promoter 850 region was analyzed by ChIP. (e) Co-IP analysis of p-AKT with G6PD in the cattle muscles, Co-IP analysis of AMPK with G6PD in the cattle muscles. MT: MSTN knockout cattle group; WT: wild-type cattle group. Data presented are means ± SD. One-way ANOVA with post hoc LSD multiple comparison test. ^∗^*p* < 0.05, ^∗∗^*p* < 0.01.

The loss of MSTN affects the TGF-*β* signaling pathway. In MT cattle, the protein level of p-AKT was significantly upregulated, while the levels of p-Smad2 and p-Smad3 were downregulated, and the level of p-p38 was unchanged (Figures 4(a)–4(c)). Smad2/3 is a transcription factor that regulates gene expression. ChIP-qPCR with anti-Smad2+Smad3 monoclonal antibody further demonstrated that Smad2/3 hardly binds to the G6PD promoter region (Figure 4(d)). These results indicate that MSTN does not regulate G6PD transcription in MT cattle via Smad2/3. Co-IP results showed that p-AKT interacts with G6PD, but knockdown of MSTN does not affect their interaction (Figure 4(e)). Loss or overexpression of MSTN affects AMPK [8, 17, 18], which is further proven by the finding that phosphorylation of AMPK in MT cattle was significantly increased (Figure 4(c)). Co-IP identification showed that knockdown of MSTN enhances the interaction between p-AMPK*α*1+*α*2 and G6PD (Figure 4(e)). This result suggests that the reduction of MSTN enhances the AMPK signaling pathway and increases the production of G6PD and that MSTN affects the expression of G6PD through AMPK.

### 3.5. MSTN Inhibits AMPK Expression through Smad2/3

**Figure 5:**
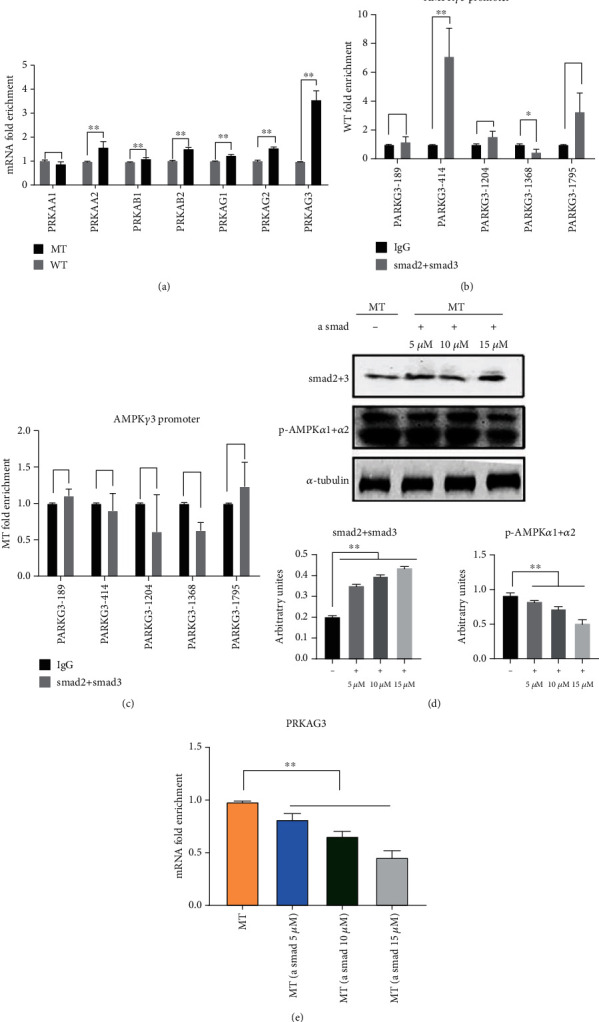
MSTN inhibits AMPK expression by Smad2/3. (a) AMPK subunit mRNA expression in muscle tissue. (b) Binding of Smad2/3 to the promoter region of the AMPK*γ*3 subunit in WT muscle tissue. (c) Binding of Smad2/3 to the promoter region of the AMPK*γ*3 subunit in MT muscle tissue. (d) Detection of p-AMPK expression in MT bovine myosatellite cells treated with Smad activator (alantolactone) for 24 h. (e) Expression of AMPK*γ*3 subunit mRNA in MT bovine muscle satellite cells treated with alantolactone for 24 h. MT: MSTN knockout cattle group; WT: wild-type cattle group. Data presented are means ± SD. One-way ANOVA with post hoc LSD multiple comparison test. ^∗^*p* < 0.05, ^∗∗^*p* < 0.01.

Loss of MSTN results in changes in AMPK subunit expression. The expression of protein kinase AMP-activated subunits PRKAA2, PRKAB1, PRKAB2, PRKAG1, PRKAG2, and PRKAG3 were significantly upregulated (Figure 5(a)). The Smad2/3 complex recruits AMPK subunit *γ*1, PARKG1-780, and AMPK subunit *γ*3, PARKG3-414 (Figures 5(b) and 5(c), S4A, S4B). MSTN knockdown did not affect the binding of the Smad2/3 complex to PARKG1-780, but the enrichment of Smad2/3 and PARKG3-414 was significantly reduced (Figures 5(b) and 5(c), S4A, S4B). The expression of Smad2+3 was upregulated by adding Smad2+3 activator in MT bovine muscle satellite cells. The results showed that upregulation of Smad2+3 inhibited p-AMPK expression (Figure 5(d)). The expression of PARKG3 subunit mRNA was consistent with the trend of p-AMPK protein expression (Figure 5(e)). Reduction of MSTN reduced the transcriptional repression of AMPK*γ*3 subunit by Smad2+3, which resulted in upregulation of AMPK.

### 3.6. MSTN Affects the Content of GSH through TGF-*β*-AMPK-G6PD

Protein sequence BLAST comparisons showed that G6PD is highly conserved, with over 90% similarity of sequences among bovines, mice, and humans (Figure S4C). A total of 19 phosphorylation sites are located in the G6PD protein sequence, 18 of which are the same in cattle, mice, and humans. Only the 490^th^ amino acid is different, which is phosphorylated tyrosine in humans and mice and valine in cattle (Figure S4C).

**Figure 6:**
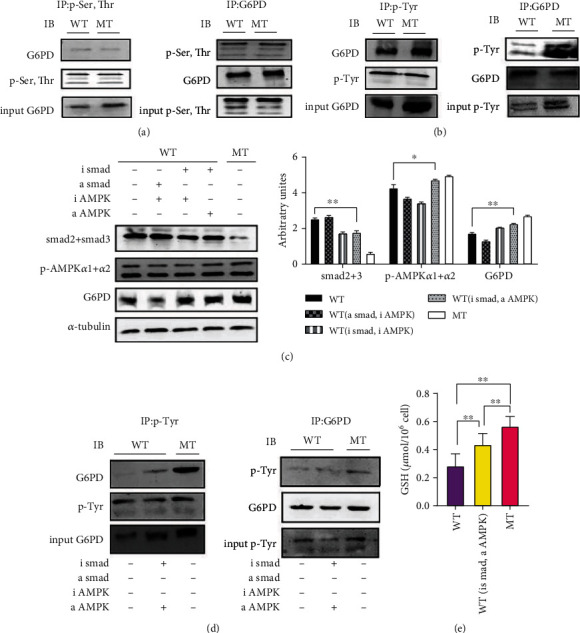
MSTN affects the content of GSH through TGF-*β*-AMPK-G6PD in cattle muscle satellite cells. (a) Co-IP analysis of G6PD Ser, Thr phosphorylation in cattle muscle. (b) Co-IP analysis of G6PD tyrosine phosphorylation in cattle muscle. (c) AMPK activator (AICAR, 300 *μ*M), inhibitor (dorsomorphin 2HCl, 5 *μ*M), Smad activator (alantolactone, 120 *μ*M), and Smad inhibitor (SIS3 HCL, 3 *μ*M). WT muscle satellite cells were treated with for 3 h. The influence of Western blot on Smad2+3, p-AMPK*α*1+*α*2, and G6PD. (d) WT muscle satellite cells were treated with AMPK activator and Smad inhibitor for 3 h. Co-IP analysis of the tyrosine phosphorine of G6PD in cattle muscle. (e) WT muscle satellite cells were treated with AMPK activator and Smad inhibitor for 3 h to determine the content of GSH. MT: MSTN knockout cattle group; WT: wild-type cattle group. Data presented are means ± SD. One-way ANOVA with post hoc LSD multiple comparison test. ^∗^*p* < 0.05, ^∗∗^*p* < 0.01.

**Figure 7:**
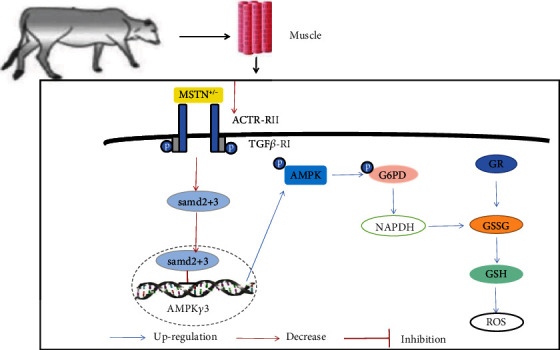
MSTN affects G6PD tyrosine phosphorylation through AMPK to improve muscle antioxidant capacity. MSTN regulates the AMPK pathway through the Smad2/3 pathway, and the upregulated AMPK activates the phosphorylation of G6PD tyrosine. The enhancement of G6PD upregulates the pentose phosphate pathway, thereby enhancing the content of nonenzymatic antioxidant GSH and improving the antioxidant capacity of muscles. In addition, the pentose phosphate pathway supplements the second stage of glycolysis to enhance its product yield.

Co-IP results showed that MT cattle have significantly higher tyrosine phosphorylation levels G6PD than WT cattle, but we observed no difference in serine and threonine phosphorylation (Figures 6(a) and 6(b)). In WT muscle satellite cells, G6PD expression levels after treatment with AMPK inhibitor and Smad activator were significantly lower than in the untreated WT cattle group (Figures 6(c)). In WT satellite cells, the expression level of G6PD in satellite cells treated with both Smad and AMPK inhibitors also showed a downward trend compared with untreated WT cattle muscle (Figures 6(c)). However, in WT satellite cells, when an AMPK activator and a Smad inhibitor were added, G6PD expression and tyrosine phosphorylation levels were significantly increased compared to the WT cattle untreated group (Figures 6(c) and 6(d)). The content of GSH in WT cattle muscle satellite cells was increased by adding an AMPK activator and a Smad inhibitor (Figures 6(e)). These results suggest that reduction of MSTN modulates the pentose phosphate pathway through the Smad-AMPK-G6PD axis, thereby increasing the antioxidant capacity of the muscle (Figure 7).

## 4. Discussion

Muscle activity increases ROS while increasing the body's antioxidant content. Antioxidants neutralize free radicals by accepting unpaired electrons, thereby inhibiting the oxidation of other molecules [26]. Antioxidant systems that remove ROS can be divided into two categories: enzymatic antioxidant systems and nonenzymatic antioxidant systems (nonenzymatic: GSH, vitamin E, vitamin C) [27]. MSTN generates ROS through Smad3, NF-*κ*B, and TNF-*α* signaling pathways [28, 29]. This study found that the lack of MSTN enhances the antioxidant capacity of skeletal muscle by upregulating the content of GSH, an antioxidant that reacts with ROS to form GSSG, thereby reducing the release of ROS [30]. However, GR mainly uses NADPH produced by the pentose phosphate pathway to provide reducing power to convert GSSG into GSH and maintain the balance of intracellular GSH [31–33].

G6PD is a rate-limiting enzyme that controls the production of NADPH. The activity of G6PD is regulated by multiple signaling pathways during transcription, posttranslation, and intracellular localization [12]. At the transcription level, G6PD activity is regulated by transcription factors Yin-Yang 1 (YY1), Sp1, CREB, TAp73, and Nrf2 [34–38]. Insulin stimulation of G6PD mRNA expression involves the PI_3_K pathway [39, 40], and arachidonic acid inhibits insulin by stimulating the AMPK and p38 MAPK pathways [41, 42]. In this study, there was no significant change in serum insulin levels, Smad2+3 did not bind to the G6PD promoter, and knocking out MSTN did not affect the binding of AKT to G6PD. T cell leukemia/lymphoma protein 1A (Tcl1) increases G6PD pre-mRNA splice and protein expression in a heterogeneous nuclear ribonucleoprotein- (hnRNPK-) dependent manner. Meanwhile, phosphatase and tensin homolog (PTEN) forms a complex with hnRNPK to inhibit G6PD pre-mRNA splice, and PTEN inactivates Tcl1 via GSK3*β*-mediated phosphorylation [25]. This experiment used qPCR analysis on GSK3*β*, and the results show that the expression of GSK3*β* is upregulated in MT cattle. However, whether GSK3*β* can regulate G6PD pre-mRNA splice and protein expression after MSTN deletion has not been determined.

AMPK is an important regulator of lipid and sugar metabolism, regulating enzyme activity through the phosphorylation of transcription factors, coactivators, and coinhibitors to achieve transcriptional regulation of metabolism, resulting in reduced anabolism and increased catabolism [40]. AMPK prevents oxidative stress by maintaining NADPH [41]. In AMPK and G6PD studies, changes in the PPP alter AMPK activity in cardiomyocytes [43]. However, 5-phosphoribulose (Ru-5-P), the product of the PPP, inhibits the LKB1-AMPK signaling pathway. Downregulation of G6PD does not affect AMPK activation [44]. In breast cancer cells, GL-V9 promotes AMPK expression and activity, leading to a decrease in G6PD [45]. This study found that mutation of MSTN enhances G6PD tyrosine phosphorylation by upregulating AMPK, leading to an increase in GSH content. The connection between MSTN and AMPK is achieved through the Smad2/3 transcription factor. Previous studies have found that MSTN reduces AMPK activity, *Glut4* gene expression, and glucose uptake and that the addition of AMPK activator AICAR reverses these effects [6]. In this study, the Smad2/3 transcription factor binds to inhibit the transcription of the AMPK*γ*3 subunit. The loss of MSTN weakens the formation of the Smad2/3 complex, thereby enhancing the expression of AMPK. After adding Smad3 inhibitor and AMPK activator to WT bovine muscle satellite cells, G6PD, tyrosine phosphate levels, and GSH content all showed an upward trend compared with untreated WT cells. These results suggest that reducing the expression of MSTN can regulate the tyrosine phosphorylation level of G6PD through the Smad-AMPK pathway to generate abundant NADPH to ensure high levels of GSH in the muscle tissue of MT cattle, thereby reducing ROS levels.

## 5. Conclusions

Inhibition of MSTN reduces the inhibitory effect of Smad on AMPK signaling, upregulates AMPK signaling, which affects G6PD activation, enhances PPP, produces more NADPH, increases the capacity of GR, and forms more reductive glutathione to remove ROS. Therefore, MSTN increases the antioxidant capacity of muscles.

## Figures and Tables

**Table 1:** Enzymatic activity in glycolysis in muscle (nmol/min/g).

Enzymatic	Group	
	WT (mean ± SD)	MT (mean ± SD)
HK	25.88 ± 7.98	42.72 ± 12.92
PFK	193.8 ± 14.30	144.10 ± 15.01
TPI	87.21 ± 4.93	48.48 ± 4.57
PK	4596.59 ± 294.59	8076.49 ± 192.88

HK: hexokinase activity; PFK: phosphofructokinase activity; TPI: triosephosphate isomerase activity; PK: pyruvate kinase activity.

**Table 2:** Products of the glycolysis pathway (mg/g).

Products	Group	
	WT (mean ± SD)	MT (mean ± SD)
FDP	6.07 ± 0.53	3.90 ± 0.33
PA	24.91 ± 4.024	37.91 ± 6.87
LA	61.17 ± 15.16	189.01 ± 16.93

FDP: fructose-1,6-diphosphate concentration; PA: pyruvate concentration; LA: lactic acid concentration.

## Data Availability

The data generated and analyzed in this study are available from the corresponding author upon reasonable request.

## Supplementary Materials

Supplementary Materials Supplementary Figure 1: knockout of MSTN alters the muscle transcriptome in cattle. Supplementary Figure 2: knockout of MSTN promotes glucose catabolism. Supplementary Figure 3: MSTN promotes muscle antioxidant capacity through G6PD in the pentose phosphate pathway. Supplementary Figure 4: MSTN affects the content of GSH through TGF-*β*-AMPK-G6PD. Supplementary Table S1: q-PCR primer sequences. Table S2: ChIP-qPCR primer sequences.
