# Supplementary material for: Myostatin Deficiency Enhances Antioxidant Capacity of Bovine Muscle via the SMAD-AMPK-G6PD Pathway
Source: Oxid Med Cell Longev. 2022 May 25;2022:3497644. doi: 10.1155/2022/3497644 (PMC9159831; doi:10.1155/2022/3497644)
Supplement: Supplementary Materials — Supplementary Figure 1: knockout of MSTN alters the muscle transcriptome in cattle. Supplementary Figure 2: knockout of MSTN promotes glucose catabolism. Supplementary Figure 3: MSTN promotes muscle antioxidant capacity through G6PD in the pentose phosphate pathway. Supplementary Figure 4: MSTN affects the content of GSH through TGF-β-AMPK-G6PD. Supplementary Table S1: q-PCR primer sequences. Table S2: ChIP-qPCR primer sequences. [file 3497644.f1.zip › Supplementary Information.docx]

**Supplementary Information**

**Supplementary Figure 1**

**
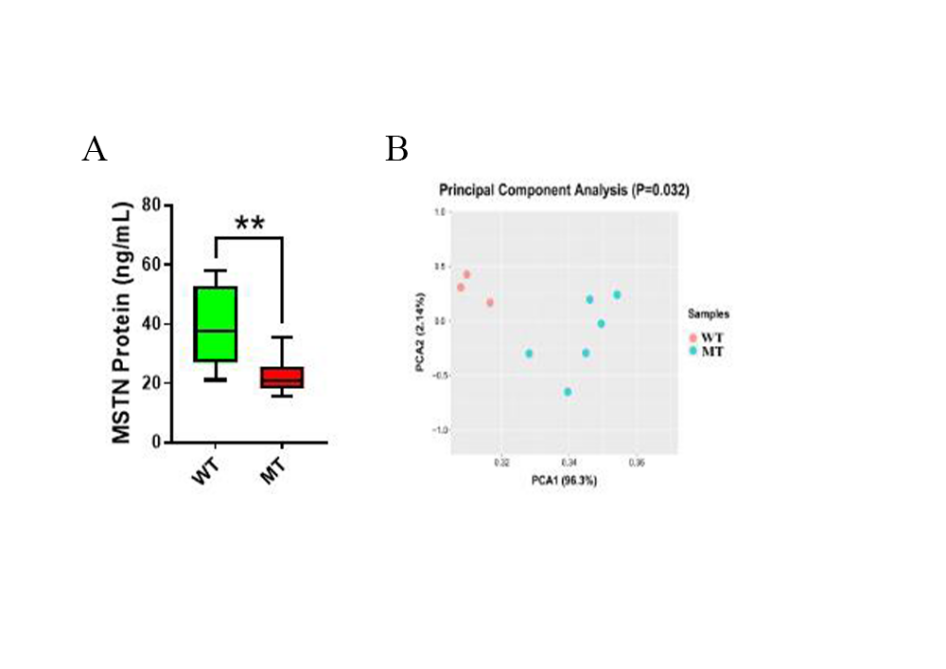
**

Supplementary Figure 1. Knockout of MSTN alters the muscle transcriptome in cattle.

(a) The MSTN protein in the plasma; (b) Cluster analysis of differential gene expression. MT: MSTN knockout cattle group; WT: Wild-type cattle group. Data presented are means ± SD. One-way ANOVA with post hoc LSD multiple comparison test. ∗*p* < 0.05, ∗∗*p* < 0.01.

**Supplementary Figure 2**


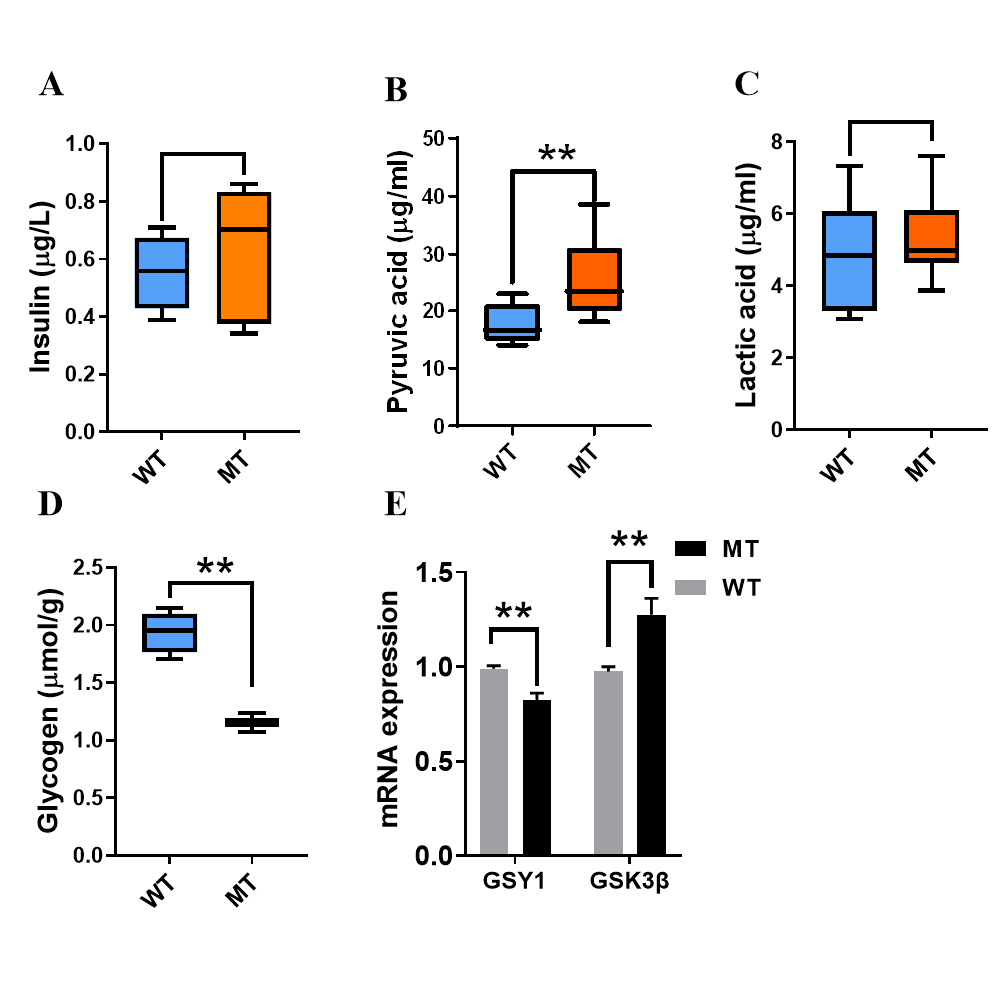


Supplementary Figure 2. Knockout of MSTN promotes glucose catabolism.

(a) Concentration of insulin in the plasma; (b) Concentration of PA in the plasma; (c) Concentration of LA in the plasma; (d) Concentration of glycogen in the muscles; (e) GSY1 and GSK3β mRNA expression in muscle. MT: MSTN knockout cattle group; WT: Wild-type cattle group. Data presented are means ± SD. One-way ANOVA with post hoc LSD multiple comparison test. ∗*p* < 0.05, ∗∗*p* < 0.01.

**
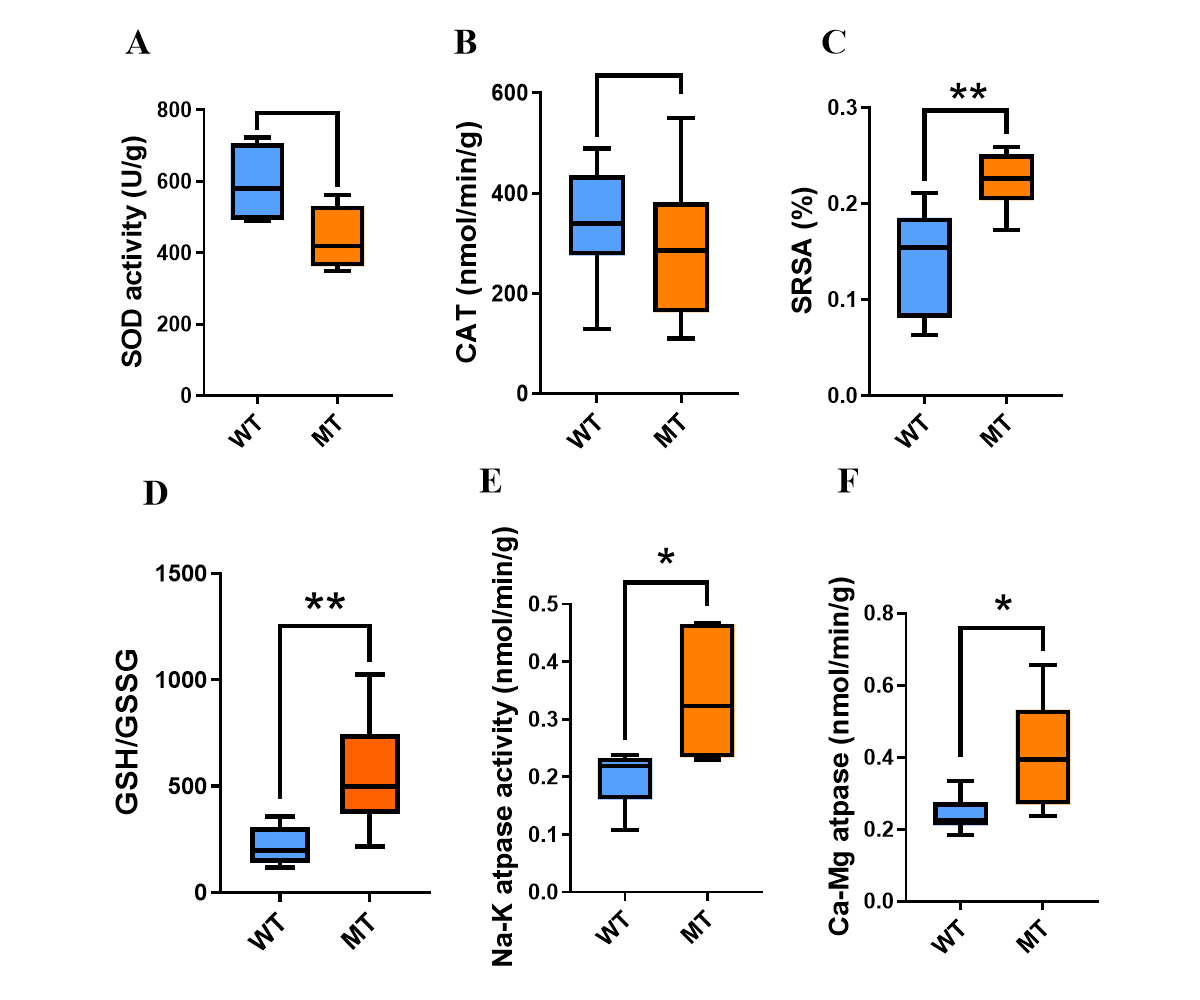
**

**Supplementary Figure 3**

Supplementary Figure 3. MSTN promotes muscle antioxidant capacity through G6PD in the pentose phosphate pathway.

(a) Activity of SOD in the muscles; (b) Activity of CAT in the muscles; (c) Activity of superoxide radical scavenging (SRSA) in the muscles; (d) Ratio of GSH/GSSG in the muscles; (e) Activity of Na^+^-K^+^ ATPase in the muscles; (f) Activity of Ca^2+^-Mg^2+^ ATPase in the muscle. MT: MSTN knockout cattle group; WT: Wild-type cattle group. Data presented are means ± SD. One-way ANOVA with post hoc LSD multiple comparison test. ∗*p* < 0.05, ∗∗*p* < 0.01.

**Supplementary Figure 4**


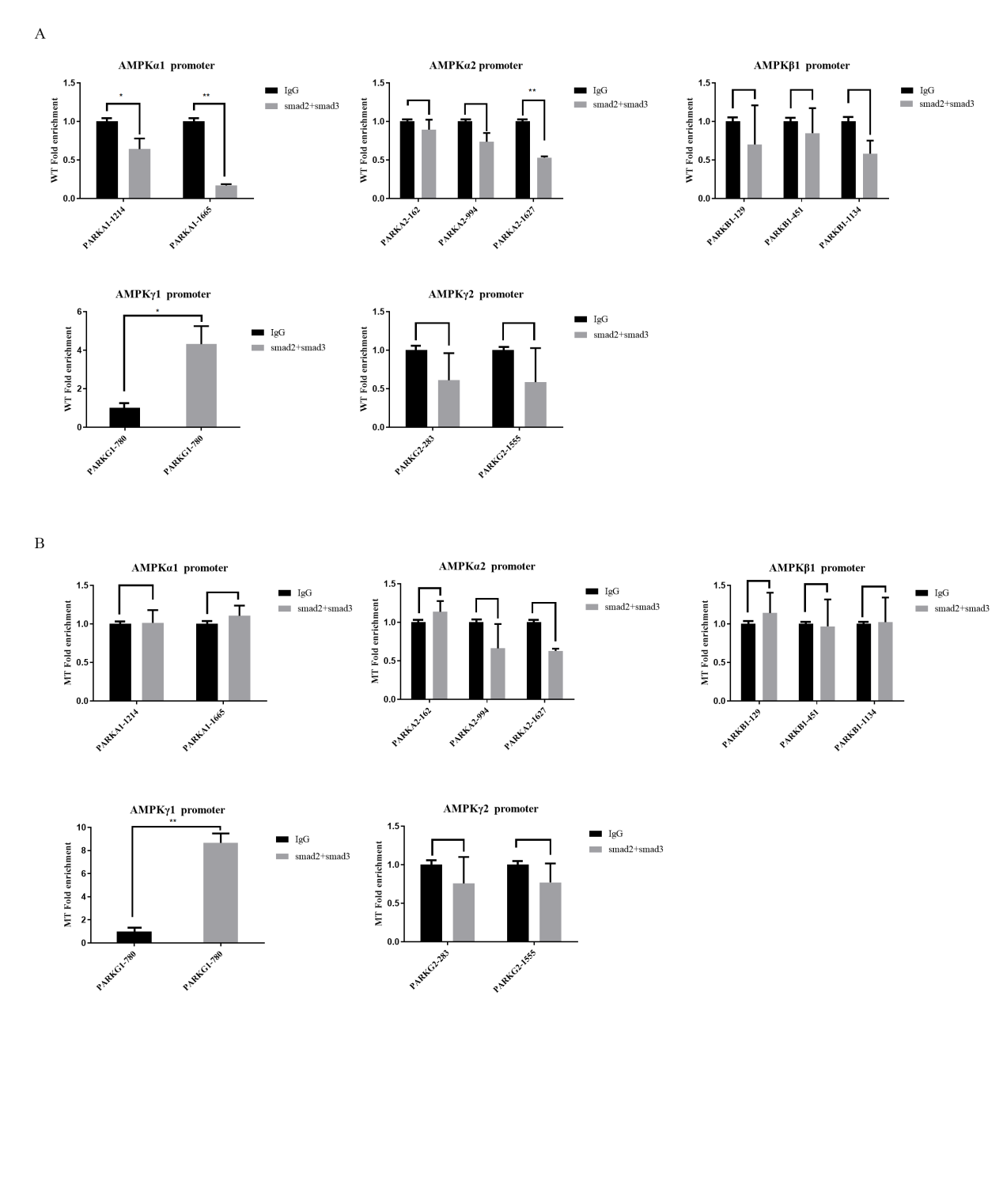


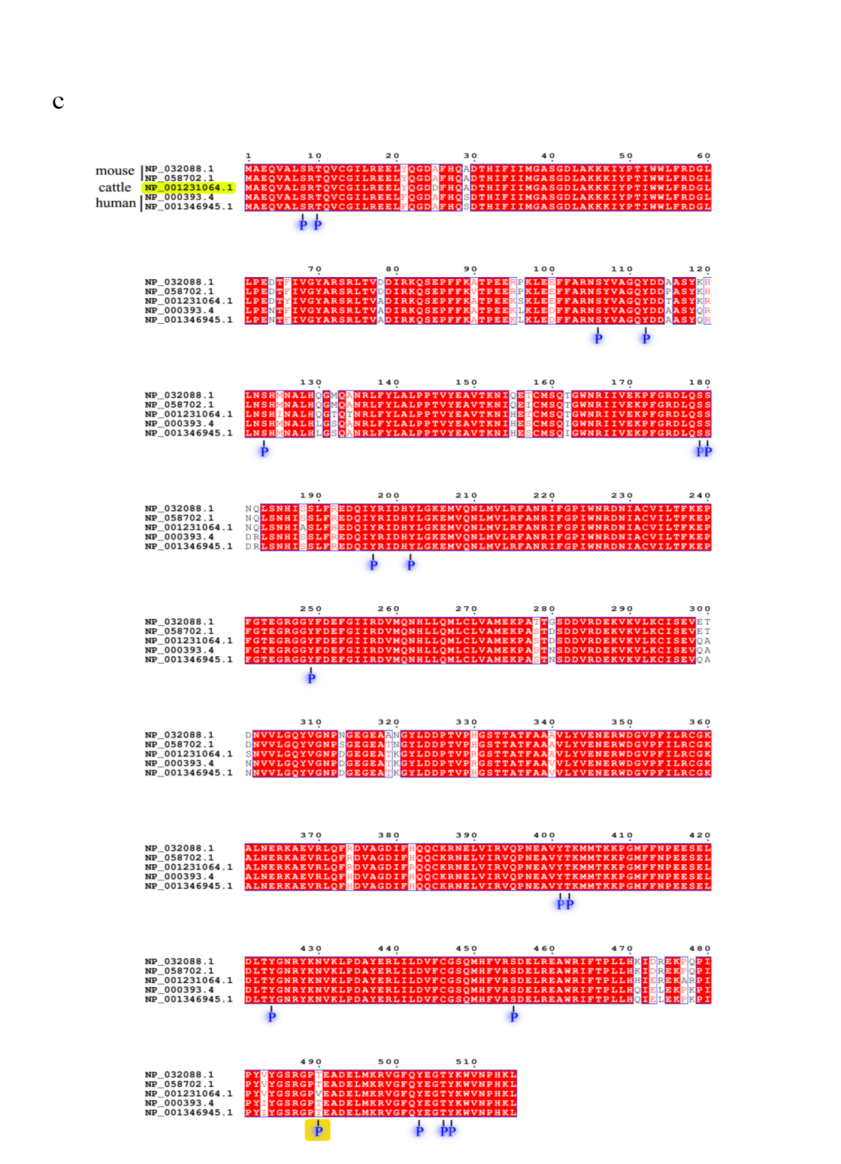


Supplementary Figure 4. MSTN affects the content of GSH through TGF-β-AMPK-G6PD.

(a) Binding of Smad2/3 to the promoter region of the AMPKα,β,γ subunit in WT muscle tissue; (b) Binding of Smad2/3 to the promoter region of the AMPKα,β,γ subunit in MT muscle tissue; (c) Mouse, bovine, and human G6PD amino acid sequence alignment results. MT: MSTN knockout cattle group; WT: Wild-type cattle group. Data presented are means ± SD. One-way ANOVA with post hoc LSD multiple comparison test. ∗*p* < 0.05, ∗∗*p* < 0.01.

Table

Table S1. q-PCR primer sequences

| Primer name | Sequence |
| --- | --- |
| PCNAF  PCNAR  G6PDF | 5’-TCCGCCTCTCGCTGTCATCC-3’  5’-TTCACGCCACTTGAGCTGATGTC-3’  5’-GACTGTCTATGAGGCTGTCACCAA-3’ |
| G6PDR | 5’-TCTCCACGATGATGCGGTTC-3’ |
| PGDF | 5’-ATGGTGCACAACGGCATAGAGTA-3’ |
| PGDR | 5’-AATCAGGAATGAGTCCAGCTCTGTC-3’ |
| PGLSF | 5’-GGCTGAGGACTACGCCAAGAA-3’ |
| PGLSR | 5’-CACCCAGAATCAGCAGGTCAA-3’ |
| PRKAA1F | 5’-GCAAGGCCACCAATGTAAGA-3’ |
| PRKAA1R | 5’-AGATGGTGTACTGATGACCTGG-3’ |
| PRKAA2F | 5’-TCGCAGTTTAGATGTTGTTGGAA-3’ |
| PRKAA2R | 5’-CCATCTCTTCAACCCGTCCA-3’ |
| PRKAB1F | 5’-AGTTGGTGGGTTCACGTCG-3’ |
| PRKAB1R | 5’-TCACTAGGTACCAGAGCCCG-3’ |
| PRKAB2F | 5’-CATGCTCCGGGTAAGGAACA-3’ |
| PRKAB2R | 5’-TGTGTGGGCTTTACGGAGTC-3’ |
| PRKAG1F | 5’-CCCCAAGCCAGAGTTCATGT-3’ |
| PRKAG1R | 5’-AGTAGATGTCCACCACACGC-3’ |
| PRKAG2F | 5’-ATGCTGATCCGTGTCGTCC-3’ |
| PRKAG2R | 5’-CAAGCTTGGAGCTGGTTGGA-3’ |
| PRKAG3F | 5’-GCACTGTGTGGGTCACTCTT-3’ |
| PRKAG3R | 5’-AGGCTTATTTCTGCGCTGGT-3’ |

Table S2 Chip-qPCR primer sequences

| Primer name | Sequence |
| --- | --- |
| G3-189F | 5’-TGTTATAGGGAGCCATTCCAGT-3’ |
| G3-189R | 5’-TGTTGGTTGGATGTGAGGGAAA-3’ |
| G3-1368F | 5’-ACACACACAGTGGCATGAGG-3’ |
| G3-1368R | 5’-GGTTTGTGGGTGGTACTGGG-3’ |
| G3-1795F | 5’-GGTCCTTCTCTCCCATCATCG-3’ |
| G3-1795R | 5’-ACTGTATCTGGGCAGCACTA-3’ |
| G3-1204F | 5’-TGGAGCATCCATTCACTCCAT-3’ |
| G3-1204R | 5’-CTGTGTGTGTTGTTCCTGCG-3’ |
| G3-414F | 5’-TTACATTCGCGGAAACACGC-3’ |
| G3-414R | 5’-TGGGCTGGCACTCACATAAG-3’ |
| B1-129F | 5’-TGTCTGCTCATGTTTCCAATCC-3’ |
| B1-129R | 5’-CCTCTTTTGTGAAACGGGGA-3’ |
| B1-1134F | 5’-ATGGACAAAGTGCATGCTCAG-3’ |
| B1-1134R | 5’-TCTTTTACTATGGCCGGTGCT-3’ |
| B1-451F | 5’-GGAAACCAGGTGACAGTTCCT-3’ |
| B1-451R | 5’-TGCCTACAGAAAAACACATGC-3’ |
| G2-1555F | 5’-TCAAAGCGCATCCCACCAAA-3’ |
| G2-1555R | 5’-GTGTCAGACCCCGGAAACC-3’ |
| G2-283F | 5’-TGAAGGCCCACCATTGTCTC-3’ |
| G2-283R | 5’-CAGGTGTGGAGTTTCTCGCT-3’ |
| G2-1441F | 5’-CCATTTCCTCCGCCGTCTTA-3’ |
| G2-1441R | 5’-CACGTGTGTCTGGAAAACCG-3’ |
| A1-1214F | 5’-TTGGTTGATGCAACTGTAAGCC-3’ |
| A1-1214R | 5’-CTGAAACGTCCTCCCCACAA-3’ |
| A1-1665F | 5’-GGCAAGGTGGTATCTCCAGC-3’ |
| A1-1665R | 5’-TCCTTTCCTCTGGCGAAGTC-3’ |
| A2-994F | 5’-AGCTCTGCCACAATTTCTATGC-3’ |
| A2-994R | 5’-CAAAGTTCCCCAATCAGTCCCA-3’ |
| A2-162F | 5’-TCCACCAATAGTTAAGACACTGC-3’ |
| A2-162R | 5’-CTAGCCGGCAATTGGAGGAA-3’ |
| A2-1627F | 5’-CCATCTCTTTCCACTCGCCA-3’ |
| A2-1627R | 5’-AAGGATGTCGCCTCGCTAC-3’ |
| G1-780F | 5’-CCTCTCTGGTAGGGAGCCAT-3’ |
| G1-780R | 5’-ACAACAGCGACCGTGC-3’ |
| G6PD-850F | 5’-ATACGAGGTCCGGGAGACAT-3’ |
| G6PD-850R | 5’-GGGTTTGTTTCCGCCTCTCT-3’ |
