# Supplementary figures and images for: Myostatin Deficiency Enhances Antioxidant Capacity of Bovine Muscle via the SMAD-AMPK-G6PD Pathway
Source: Oxid Med Cell Longev. 2022 May 25;2022:3497644. doi: 10.1155/2022/3497644 (PMC9159831; doi:10.1155/2022/3497644)

**A**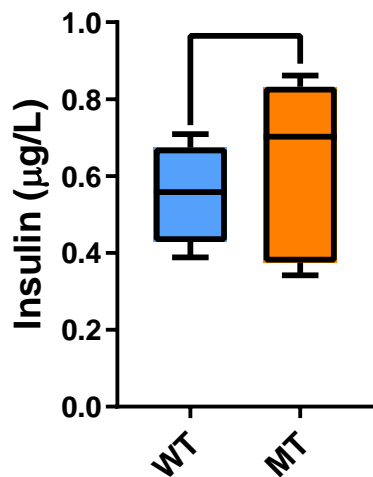**B**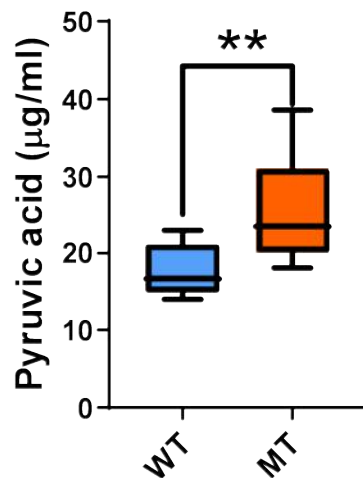**C**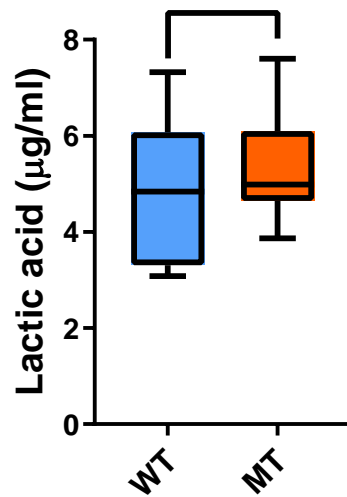**D**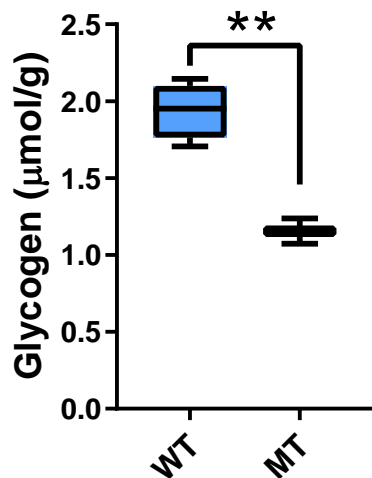**E**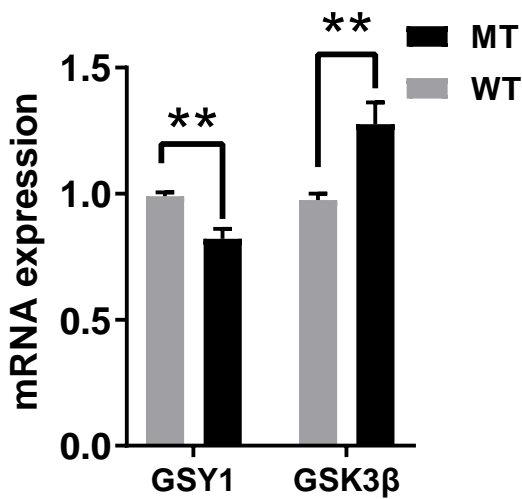

Supplement: Supplementary Materials — Supplementary Figure 1: knockout of MSTN alters the muscle transcriptome in cattle. Supplementary Figure 2: knockout of MSTN promotes glucose catabolism. Supplementary Figure 3: MSTN promotes muscle antioxidant capacity through G6PD in the pentose phosphate pathway. Supplementary Figure 4: MSTN affects the content of GSH through TGF-β-AMPK-G6PD. Supplementary Table S1: q-PCR primer sequences. Table S2: ChIP-qPCR primer sequences. [file 3497644.f1.zip › Supplementary Figure2.pdf]

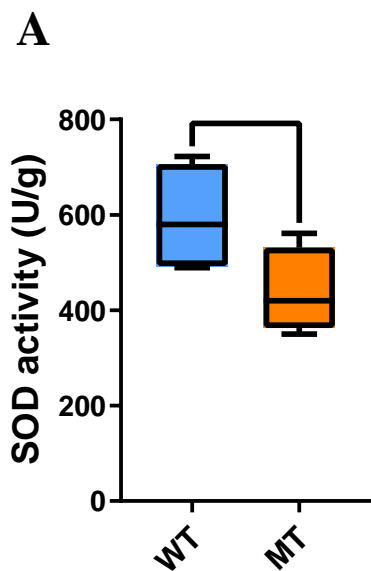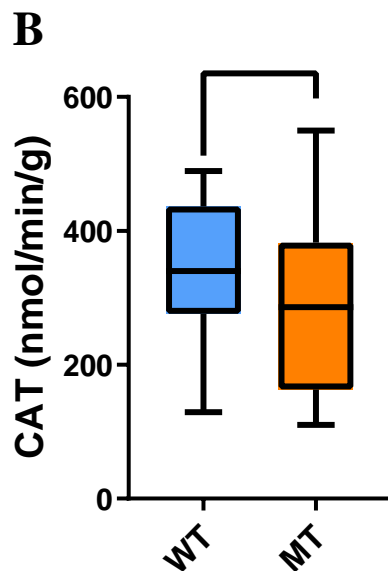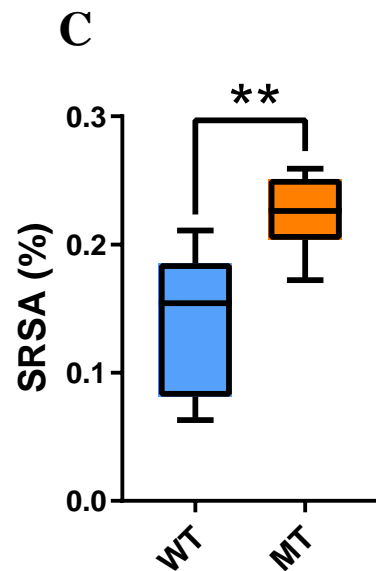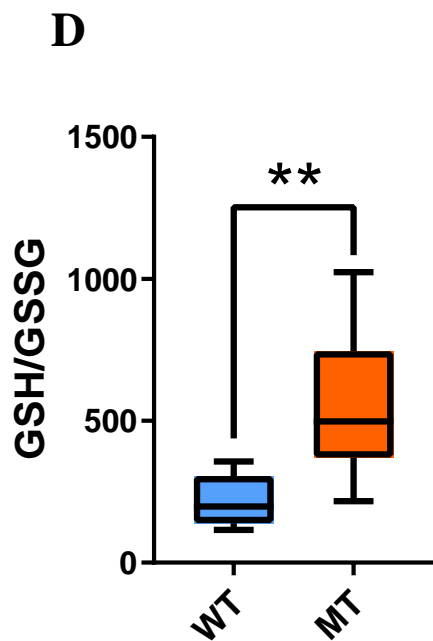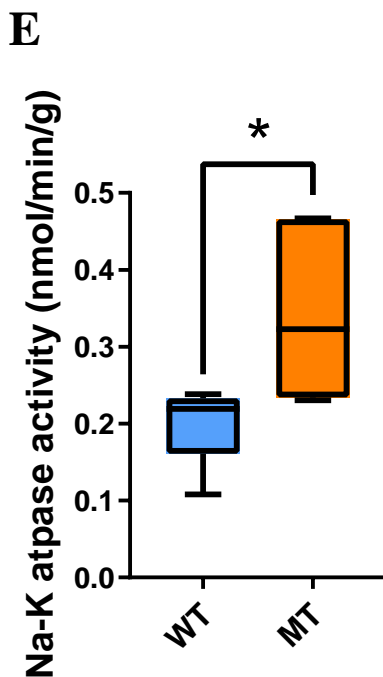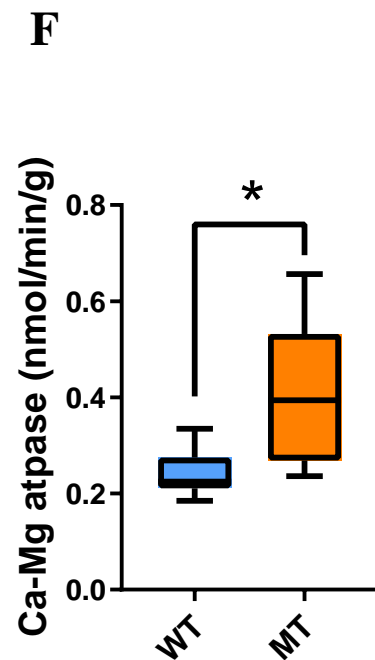

Supplement: Supplementary Materials — Supplementary Figure 1: knockout of MSTN alters the muscle transcriptome in cattle. Supplementary Figure 2: knockout of MSTN promotes glucose catabolism. Supplementary Figure 3: MSTN promotes muscle antioxidant capacity through G6PD in the pentose phosphate pathway. Supplementary Figure 4: MSTN affects the content of GSH through TGF-β-AMPK-G6PD. Supplementary Table S1: q-PCR primer sequences. Table S2: ChIP-qPCR primer sequences. [file 3497644.f1.zip › Supplementary Figure3.pdf]
